# Biological Mechanisms of Pain Management in Lumbar Disk Herniation: Focus on Cytokine Correlations and Therapeutic Approaches

**DOI:** 10.3390/ijms262210830

**Published:** 2025-11-07

**Authors:** Karla Rožac, Anita Matić, Dino Budrovac, Dijana Hnatešen, Ivan Radoš, Kristina Kralik, Martina Smolić, Tanja Kovač Lukić

**Affiliations:** 1 Department of Anatomy, Histology, Embryology, Pathological Anatomy and Pathological Histology, Faculty of Dental Medicine and Health, Josip Juraj Strossmayer University of Osijek, 31000 Osijek, Croatia; krozac@fdmz.hr (K.R.); tkovac@fdmz.hr (T.K.L.); 2 Department of Pathophysiology, Physiology and Immunology, Faculty of Dental Medicine and Health Osijek, Josip Juraj Strossmayer University of Osijek, 31000 Osijek, Croatia; anitamatic@fdmz.hr; 3 Faculty of Medicine Osijek, Josip Juraj Strossmayer University of Osijek, 31000 Osijek, Croatia; dino.budorvac@gmail.com (D.B.); hnatesen@yahoo.com (D.H.); irados11@gmail.com (I.R.); kristina.kralik@gmail.com (K.K.); 4 Department of Anaesthesiology, Reanimatology and Intensive Care, University Hospital Centre Osijek, 31000 Osijek, Croatia; 5 Nursing Institute “Professor Radivoje Radić”, Faculty of Dental Medicine and Health Osijek, Josip Juraj Strossmayer University of Osijek, 31000 Osijek, Croatia; 6 Department of Pharmacology and Biochemistry, Faculty of Dental Medicine and Health Osijek, Josip Juraj Strossmayer University of Osijek, 31000 Osijek, Croatia

**Keywords:** inflammation, inflammatory biomarkers, low back pain, lumbar disc herniation, ODI, SF-MPQ, therapeutic approach

## Abstract

Lumbar disk herniation is a common cause of pain in people older than 30, often associated with workload, where the therapeutic approach includes different methods of treatment; therefore, the aim of the study was to inspect the effectiveness of different methods of treating pain caused by lumbar disk herniation in relation to pro-inflammatory and anti-inflammatory parameters before and after two weeks of therapy. There were twenty-eight participants with a diagnosis confirmed by a specialist who also assigned the participant to a clinically appropriate type of treatment. Pain and disability were assessed using the SF-MPQ and ODI (Title: Immune Response During the Conservative and Minimal Invasive Treatment of Pain Caused by Lumbar Disc Herniation, Clinical Trials Number (NCT06545812), Initial Release 23 July 2024, Last Release 27 August 2025). In addition to the above questionnaires, serum samples were collected before and after therapy for analysis of inflammatory biomarkers. Although there was no statistically significant difference, the tendency of decreases in IL-1 beta and IL-8 in the median levels (interquartile range) was observed after conservative treatment. The results indicate role of inflammatory mechanisms in the treatment of disk herniation and support the benefits of a conservative approach through the regulation of pain, disability, and cytokine activity.

## 1. Introduction

Low back pain is one of the most common causes of disability and a substantial burden on the health care system. It could therefore be said that it is a comprehensive psychophysical problem that affects not only the individual but also the social community [1]. Pain can be classified according to its duration as acute or chronic, which are closely related to anatomical changes, while according to etiology, pain can be nociceptive or neuropathic [2,3,4]. Nociceptive pain is caused by actual or potential damage to non-nervous tissue, is usually clearly localized, and can be confirmed diagnostically by the presence of damage or pathology that corresponds to the clinical presentation of pain. For example, annular fissures in degenerative disks often contain granulation tissue, which can attract and cause the ingrowth of nerves capable of transmitting nociceptive signals. These can release inflammatory mediators and increase expression of molecules such as nerve growth factor and its receptors [5,6]. In contrast, neuropathic pain results from a lesion or disease of the somatosensory nervous system and is often accompanied by sensory symptoms such as tingling, numbness, or weakness, with the pain being distributed according to a neuroanatomically expected pattern. Common phenomena include hyperalgesia and allodynia [6,7]. Nociplastic pain, on the other hand, occurs as a consequence of altered nociception, often affects areas that are not neuroanatomically logical, and is accompanied by the appearance of symptoms such as fatigue, sleep disturbances, and cognitive difficulties. The findings of tissue damage usually cannot fully explain the clinical picture [6].

As already mentioned, the pain that occurs and has the possibility of migrating is caused by pathological changes, which include lumbar disk herniation (LDH). This can be caused by some type of traumatic injury, degenerative changes, and biomechanical compression forces [2,8].

The morphology of LDH has been well described in terms of extent of the disk space affected by bulging, protrusion, extrusion, and sequestration of the disk [4]. The intervertebral disk (IVD) is considered an immunologically privileged organ. This is due to its unique structure in which the inner gelatinous part (nucleus pulposus) isolates it from the host immune system, where the outer connective ring (annulus fibrosus) and the cartilaginous end plate, together with factors that cause molecular immunosuppression, form a blood–nucleus pulposus barrier [4,9,10]. The pathological process occurs when the nucleus pulposus triggers an immune response that is consequently activated when the blood–nucleus pulposus barrier is damaged. Then, the nucleus pulposus becomes fibrotic due to significant loss of proteoglycans. At this time, there is an increase in inflammatory mediators that consequently stimulate the creation of discogenic pain, which increases the pathogenic process and leads to aging and autophagy [4,10]. Disruption of the blood–nucleus pulposus barrier and exposure of the microenvironment to immune responses consequently triggers an autoimmune response responsible for multiple pathological mechanisms of blood vessel formation in diseased tissue (neovascularization) and accumulation of immune cells in the tissue (infiltration) [4,9,11,12].

Immune cells that secrete inflammatory cytokines such as interleukin-1β (IL-1β) and tumor necrosis factor-α (TNF-α) actually try to regulate matrix metalloproteinases (MMPs), accelerating the resorption of the extracellular matrix and the change in immune cells such as macrophages, T cells (CD4+, CD8), neutrophils, and mast cells that release cytokines; these cells have the ability to migrate and thereby promote inflammation [13,14]. CD4+ T cells are divided into two subtypes: the first type (Th1) enhances cellular resistance by producing interferon gamma (IFN-γ) and IL-2, and the second type (Th2) comprises the humoral immune response that enhances the expression of interleukins (IL-4, IL-5, IL-6, IL-10, and IL-13) [4,5,9,13,14]. Although the details of how these processes occur are not yet fully understood, cells within the disk begin to produce increased amounts of inflammatory molecules such as IL-1, TNFα, IL-6, IL-8, and IL-17 [11]. For example, in people suffering from sciatica, the presence of various inflammatory and anti-inflammatory proteins such as interleukins IL-1β, IL-6, IL-8, and TNF-α, which are known for their role in inducing and regulating inflammation, has been detected in their blood, cerebrospinal fluid, and tissue samples [11,12]. These molecules create an unfavorable environment that accelerates tissue breakdown. IL-1 in particular stands out, playing an important role in initiating these harmful changes during disk degeneration. Although some other cytokines and chemokines, such as Monocyte chemoattractant protein-1 (MCP-1), TNFα, and IL-8, may not act directly on the disk cells themselves due to a lack of receptors, they can still exit the disk and spread to surrounding tissues where they can cause additional inflammation, attract immune system cells after the outer layer of the disk and endplates rupture, and increase the sensitivity of nerve endings causing pain [11,14,15].

Accordingly, it is essential to understand the immunologically privileged organ and the inflammatory processes in order to choose the right treatment approach [9,14]. Although lumbar disk herniation (LDH) is treated conservatively in most cases, which is the therapeutic approach of first choice for mild motor difficulties or a transitional period; treatment is primarily aimed at controlling painful symptoms. Despite expressed radicular symptoms, extrusion and sequestration forms of herniation often show a tendency for spontaneous regression compared with protrusion and bulging types, which is attributed to their exposure to systemic circulation and consequently activation of resorptive mechanisms [3]. However, when conservative therapy does not lead to satisfactory clinical improvement or there is progression of neurological symptoms but therapy is unsuccessful, minimally invasive treatment methods are indicated, and the final treatment option is surgery. Surgical intervention is indicated only in cases of progressive muscle weakness accompanied by progressive neurological deficits and is used with caution, most often in the context of cauda equina syndrome [14,15,16].

The guidelines of the American Society of Interventional Pain Physicians (ASIPP), published in 2021, recommend LDH treatment methods according to the stage of the disease [14]. They recommend a conservative treatment approach that includes physical therapy (spinal manipulation, exercises, mechanical traction, electrotherapy—interferential current (IFC) and transcutaneous electro neurostimulation (TENS), ultrasound, laser, magnetotherapy, and orthoses) and forms of acupuncture (classical, pharmaco-acupuncture and electro-acupuncture). In cases of chronic pain, the guidelines recommend minimally invasive epidural interventions—caudal, interlaminar, and transforaminal approaches—with the highest level of evidence of effectiveness. In particular, transforaminal epidural steroid injection (ESI TF) has been highlighted as the most effective method for reducing inflammation, protecting nerve fibers, and relieving pain. In the context of treatment, pain from lumbar disk herniation is aimed at controlling inflammation, reducing nociceptor hypersensitivity, and modulating neuronal plasticity, which allow the use of anti-inflammatory drugs, nerve blocks, physical therapy, and neuroplastic interventions. [13,17,18,19]. Therefore, the aim of this study was to evaluate the effectiveness of different methods of treatment of pain caused by herniated intervertebral disk in the lumbar region and the level of inflammatory and anti-inflammatory response before and after treatment, as well as measuring the level of pain through the Short Form McGill Pain Questionnaire (SF-MPQ) and the Oswestry Low Back Pain Questionnaire (ODI).

## 2. Results

The study included 28 participants diagnosed with lumbar disk herniation. The analysis showed differences in median age between treatment groups: in the conservative treatment group, the median age was 59.5 years (interquartile range, 55 to 67), while in the minimally invasive treatment group, the median age was 52 years (interquartile range, 39 to 61).

Most of participants are female 21 (75%), and their marital status is married 20 (71%). Out of 28 participants, 21 (75%) have completed secondary vocational education. Furthermore, 11 participants (39%) are employed, while 9 (32.1%) are retired. Although the average age of the respondents were working-age, three (11%) of them were on long-term sick leave and not employed due to functional limitations caused by pain symptoms (Table 1).

The SF-MPQ examined the type of pain experienced in the past week, current pain, i.e., sensory and affective domains, and included a visual analog scale (VAS) for the subjective assessment of pain by the participants. The questionnaire is intended for the examination of chronic pain in patients and for this reason was used to compare pain before (baseline) and after (after two weeks) two different therapies (conservative and ESI TF).

There were no significant differences between groups and between measurements in the sensory and affective domains of the SF-MFQ, although a certain decrease can be observed according to the median (interquartile range). However, there was a statistically significant difference in pain in the past week between measurements (Wilcoxon test, *p* = 0.02) and in both groups (Mann–Whitney U test *p* = 0.04), indicating the effect of the therapy.

Before both therapies, the pain was significantly more severe in relation to other measurements in both groups. Among participants who underwent conservative treatment, current pain values were significantly between measurements (Wilcoxon test, *p*= 0.01) (Table 2).

**Figure 1:**
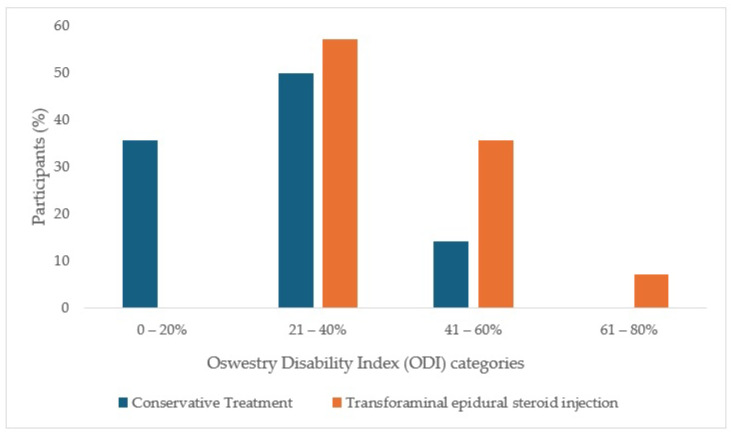
Oswestry disability index (ODI) categories in patients treated with conservative therapy or trans-foraminal epidural steroid injection at baseline and after two weeks.

Among the ESI TF group, after treatment, there was a significant improvement according to the ODI in the level of functioning and activities of daily living (Wilcoxon test, *p* = 0.008); moreover, there was a statistically significant difference between groups in baseline (Mann–Whitney U test *p* = 0.006) (Table 3). Furthermore, there was also difference in the disability index between groups in baseline (Fisher’s Exact Test, *p* = 0.04) (Figure 1).

There were no significant differences between groups and between measurements in cytokine levels, but a decrease in cytokines was observed in the group of participants who underwent conservative treatment. Although there is no statistically significant difference, a tendency decreases in the Median (interquartile range) can be noticed after conservative treatment in IL-1 beta and IL-8 (Supplementary Table S1).

Spearman’s correlation coefficient was used to appraise the relationship between cytokine levels in both groups and both measurements. In the group of participants who underwent conservative treatment, there was negative correlation between TNF-α and IL-6 in baseline (Rho = −0.712; *p* = 0.006), and also a positive correlation between IL-1β and IFN-γ after 2 weeks of therapy (Rho = 0.587; *p* = 0.03). On the contrary, participants who underwent a minimally invasive treatment method presented a positive correlation between IL-1β and IFN-γ in the baseline (Rho = 0.772; *p* = 0.001) (Table 4).

Both outcome measurements showed that there was a positive correlation between IFN-γ and current pain intensity in participants who underwent a minimally invasive treatment method before the therapy (Rho = 0.561; *p* = 0.04) (Supplementary Table S2) and total ODI after 2 weeks of therapy in the same participants (Rho = 0.582; *p* = 0.03) (Supplementary Table S3). Comparison of other correlations in both tables did not reveal a statistically significant difference.

## 3. Discussion

Lumbar disk herniation is a commonly known burden on the socioeconomic system due to prolonged absence from work, expensive diagnostics and therapy, and long-term problems [20,21,22,23].

As Yan et al. [24] mentioned, this trend will increasingly result from the modern lifestyle and work environment that includes insufficient movement and prolonged sitting and standing time depending on the type of work, which consequently leads to the appearance of pain symptoms. Previous research by Kögl et al. [21] indicates an increasing prevalence of episodes of painful symptoms in the lumbar region among the adult population. Clinically, pain typically radiates from the lumbar region along the sciatic nerve into the leg, while involvement of the upper lumbar segments may result in painful projection along the femoral nerve. These symptoms are associated with direct health care costs, primarily pharmacological treatment, while secondarily leading to reduced work capacity and productivity, potential development of disability, and consequent economic burden [21,24].

Current treatment modalities, as already mentioned, still show limitations such as insufficiently supported scientific research with objective parameters on the effectiveness of therapy in reducing pain [25]. In accordance with the above challenges, this study provides insight into the benefits of different methods of treating pain caused by lumbar disk herniation, as well as the associated immune response before and after therapy.

In this study, although we were talking about small number of participants involved, the median score was 52 to 59.5, which was still the working population; although a small number of subjects were shown to be on sick leave/long-term sick leave due to the same pain, it is important to emphasize that some of the subjects were forced to retire because of it.

Today, pain is one of the main and leading symptoms that can cause motor and sensory deficits and consequently cause a weakened response, which was shown with degree of pain in our study before the same therapy, where we also monitored the effect of therapy after 10 days of therapy and achieved a statistically significant difference (*p*^†^ < 0.02), in parallel with the study conducted by Satpute et al. (2018), which reported a reduction in leg and back pain in patients after exercise and electrotherapy, as well as less disability and overall satisfaction in the short and long term [26]. Although the VAS was part of the SF-MPQ in patients undergoing ESI TF, there were no statistically significant differences, as a similar study showed, however, over a period of one month and three months, a reduction in pain, but this can be explained by the fact that local anesthetics reduce local inflammation and steroids have an anti-inflammatory effect, where the patient’s effect can be several months to enable physical therapy, and it can be very short-lived, only a few days or no effect at all [26,27].

Prospective randomized research conducted by Budrovac et al. [22] examined the effect of epidural steroid injection on discoradicular contact through several questionnaires, and one of the questionnaires was the ODI, which showed that the subjects at the beginning of the study had a higher total score than one month after the therapy, which can also be compared with the conducted research in which the total score was higher before the therapy and decreased after 2 weeks of the therapy and at the same time showed a statistically significant difference. The aforementioned result confirms the subjective improvement in the patients’ condition after the therapy itself [22].

Similarly to previous studies, conservative treatment was proven effective in treating patients with lumbar disk protrusion, indicating that the therapy itself has an effect on reducing pain, improving mobility, and reducing symptoms in general. Various methods of conservative treatment have been studied in the past few years in patients with protrusion of the disk in lumbar part. The most commonly mentioned treatment includes patient education, back school, exercises, physiotherapy, behavioral therapies, balneotherapy, and electrotherapy (ultrasound, TENS, IFC, laser) [20,28,29].

In a study by Tarcău et al. [29], they compared the final assessment results between two groups, one of which received only electrotherapy and the other an experimental group that received a physical therapy program that included both hydrotherapy and electrotherapy. They found that there were significant differences between the groups in terms of pain perception as measured by the SF-MPQ (*p* < 0.05), ODQ score (*p* < 0.05), and ODQ (%) (*p* < 0.05). The program included complex rehabilitation techniques to reduce pain and impairment in patients with lumbar spine disk protrusion. After six months, the experimental group enhanced more than the group that received only electrotherapy in terms of pain (VAS, SF-MPQ), disability (ODQ, ODQ%), and LS score (*p* < 0.05) [29], which supports the conducted research because it showed a reduction in pain perception after just 2 weeks of therapy. Looking at objective components such as cytokine levels and observing the correlation of cytokines in groups by measurement point with SF-MPQ, no statistically significant difference was noticed in the group of patients who underwent conservative treatment, but it can clearly be seen that the current pain intensity in IL-8, which is an inflammatory cytokine, decreased.

A study conducted at Oslo University Hospital, Norway by Pedersen et al. (2015) [30] led to interesting conclusions about the association of chronic lumbar radicular pain with persistent increases in serum IL-6 and IL-8 levels. In the study, patients with more severe pain (VAS ≥ 3) 12 months after disk herniation were observed, where statistically significantly higher serum concentrations of pro-inflammatory cytokines IL-6 and IL-8 were found compared with patients with mild or absent pain (VAS < 3) (*p* ≤ 0.01), with age and smoking for IL-6 and smoking and treatment for IL-8 as additional comparison variables [30].

Furthermore, Krock et al. [31] conducted a study in 2019 in which IL-8 was elevated in the cerebrospinal fluid and disk tissue of people with disk degeneration with chronic pain compared with people with degeneration without pain. While in a mouse model, blocking IL-8 signaling contributed to the reduction in pain and degeneration, suggesting an important role for IL-8 in the development of chronic low back pain. An increase in IL-8 levels may indicate not only mechanical compression of the nerve root, but also the biochemical effect that a herniated disk has on the surrounding tissues. Although a tendency for a decrease in IL-1β and IL-8 levels was observed with conservative treatment, the follow-up was short-term. Longer terms studies would be needed to confirm these results [31,32].

Another important molecule that has a function in regulating pain and neuroinflammation is IFN-γ. Interferon gamma has a pronociceptive effect in lumbar disk herniation, as it is released during degeneration by stimulating circulating macrophages at the level of disk tissue and in the bone marrow. IFN-γ increases the excitability of dorsal horn neurons and causes long-term depolarization of inhibitory circuits, which contributes to central sensitization and enhanced pain perception [33,34,35,36].

Based on the above, Kamieniak et al. [36], in 2020, conducted a study in which they observed the correlations of IFN-γ with the assessment of pain and disability via the ODI in patients before and after lumbar microdiscectomy. Although they did not find a significant correlation between the aforementioned scale and IFN-γ levels, a slight trend approaching significance was shown, where participants had more severe complaints before the surgery and thus had higher IFN-γ levels (*p* = 0.058). Sabirov et al., 2023 [37], in their research on the comparative profiles of different cytokines in blood serum in patients with spinal cord injury (SCI), found a significant increase in IFN-γ. They concluded that after this type of traumatic injury, the inflammatory process increases indicating possible structural changes. In parallel, this study also showed a tendency for IFN -γ to change, and a positively statistically significant difference after the ESI TF treatment approach in the form of IFN-γ regulation as well as disability [36,37].

Encompassed by these cytokines, IL-1β and TNF-α are exceedingly studied. In its fundamental physiological role, TNF-α regulates immune cell functions and induces apoptotic cell death, not to mention cell differentiation and the creation of new cells during the formation of new tissue known as proliferation. Immense levels of circulating cytokines, along with TNF-α, have been observed in patients suffering from radiculopathy after disk herniation. Some studies have already shown in a rat model that TNF-α “mimics” the effects of a herniated nucleus pulposus by inducing apoptosis in dorsal nerve root ganglia, leading to an immune response [38,39,40,41].

Zu et al. [41] conducted a study examining the role of TNF-α and IL-4. What they concluded from the obtained results was that the level of TNF-α in these patients decreased during the first month of multidisciplinary treatment but remained high throughout the follow-up period. TNF-α, as a pro-inflammatory mediator, plays one of the main roles in the pathophysiology of radicular pain in the lumbar segment. The results of TNF-α in this study do not show any significant difference, but we can also attribute this to the limitation due to the small number of patients [41].

Already well-known mediators of inflammation and pain, IL-6 and TNF- α have a major impact on disk degeneration. For example, IL-6 can trigger downstream signaling pathways that will contribute to inflammation and catabolic processes within the IVD. TNF-α can exacerbate intervertebral disk (IVD) degeneration by inducing the expression of MMPs and other catabolic enzymes responsible for extracellular matrix degradation, thereby contributing to a complex inflammatory microenvironment that promotes degenerative disk changes. Although aging also contributes to changes in the IVD, the disk is additionally exposed to biomechanical stress from weight bearing and a sedentary lifestyle [25,42]. The results of this and earlier studies indicate the possible role of IFN-γ as a predictive biomarker of a favorable response to epidural steroid injections in patients with lumbar disk herniation. IFN-γ, a key inflammatory cytokine, activates macrophages and monocytes and promotes the release of inflammatory mediators, which may contribute to the maintenance of the immune response and the persistence of pain. Previous research from 2018 also highlighted locally increased expression of IFN-γ and elevated levels of IL-6, IL-8 and TNF-α in the serum, which points to a classic inflammatory process with the formation of granulation tissue rich in macrophages and T-lymphocytes, which can contribute to disk degeneration. Furthermore, the positive correlation between IFN-γ and IL-1β confirms their role in inducing and maintaining the inflammatory response in conservative group [43,44]. Then, research from 2019 indicated the expression of IL-6 in the serum and immunohistochemically indicated degenerative changes. The very next year, a study was conducted in which IL-1β significantly increased the expression of IL-6 and IL-8, vascular endothelial growth factor, chemotactic factor (MCP-1), and disk degradation factor (MMP-3) in IVD cells during hypoxia, which are key to catabolic changes promoting inflammation and degeneration. Research conducted in 2023 showed that increased expression of IL-6 and IL-8 affects the key characteristics of the patient or tissue, such as the degree of degeneration, age and level of pathology; so the above affects tissue degeneration and the development of pain [10,45,46]. Interesting conclusions were drawn in a study conducted in 2025 by Kabdesh et al. [47] who observed molecular responses after thoracic spinal cord contusion and the impact it had on the lumbar spinal cord. They discovered significant expression of TNFα in the lumbar region during the chronic phase. This response probably resulted from the inducing effects of TNFα, which stimulated increases in neurotoxic M1 microglia or macrophages and the expression of TNFα, MCP-1, and macrophage inflammatory protein 1-alpha (MIP-1α). During the chronic phase, activated microglia are more sensitive than astrocytes to damaging molecules, and early pro-inflammatory effects induce A1 astrocytes. With the progression of the pathology, they switch to the M2 phenotype and establish an interaction with A2 astrocytes [47].

Although several studies have been published on the host immune response to lumbar disk herniation and the possible expression of inflammatory parameters has been known for some time, none of these studies have compared treatment methods such as conservative and minimally invasive, which may be a predictor of future primary interventions in such patients. The key thing that this study showed was that there was definitely a positive correlation in the conservative type of treatment between IL-1β and IFN-γ after only two weeks of therapy, which can be shown to reduce the inflammatory process, while at the beginning of therapy a negative correlation was visible between TNF-α, which was expressed in relation to IL-6. In contrast, before therapy with epidural steroid injection, a positive correlation was also visible between IL-1β and IFN-γ. It is important to emphasize that the results obtained were conducted on a small sample, and it is necessary to conduct research on a larger sample.

## 4. Materials and Methods

### 4.1. Study Design

**Figure 2:**
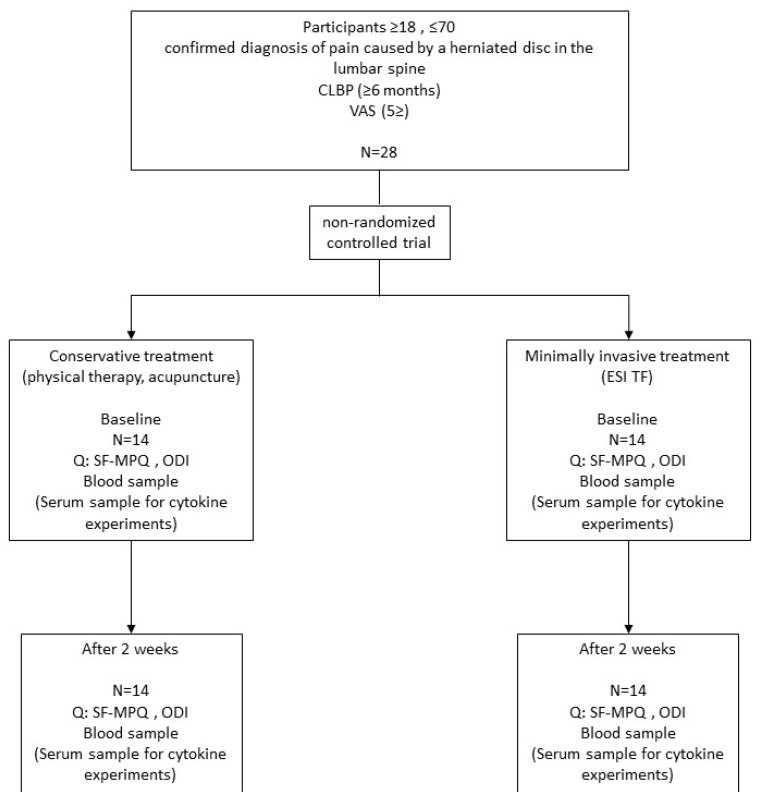
Study design.

The study encompassed 28 patients aged 18 to 70 years with a radiologically confirmed diagnosis of lumbar disk herniation in the form of protrusion or extrusion, who were admitted to the Department of Pain Management of the Clinical Hospital Center Osijek. The non-randomized controlled trial was conducted from October 2024 to February 2025 after approval by the ethics committee of the Clinical Hospital Center Osijek (No.: R1-6730/2024 dated 11 June 2024), and the Faculty of Dental Medicine and Health Care Osijek, Josip Juraj Strossmayer University of Osijek (CLASS: 602-01/24-12/02, REGISTRATION NUMBER: 2158/97-97-10-24-50 dated 3 July 2024) and in accordance with the Declaration of Helsinki. All participants signed a written informed consent form describing the program to be performed, and the program was explained orally to the patients. Patients were divided into 2 groups according to the type of treatment with conservative methods and minimally invasive methods of treatment, after signing an informed consent. Blood samples were collected at two different time points before therapy (baseline) and after two weeks from the start of therapy. Patients assessed their pain using the Short Form McGill Pain Questionnaire (SF-MPQ), and the degree of disability using the Oswestry Low Back Pain Questionnaire (ODI) (Figure 2).

### 4.2. Patients

The inclusion criteria for participants are patients of both sexes, aged 18 to 70 years, with unilateral radicular pain in the lumbar segment with a confirmed diagnosis through clinical imaging and pain duration of up to six months. Symptomatic disk herniation at one level with pain intensity measured on a VAS from 0 to 10, which is equal to or greater than 5 pain intensity along the leg. The exclusion criteria are patients younger than 18 or older than 70 years and patients with systemic, local infections in the lumbar spine and dermatological diseases in the lumbar spine; pregnancy; central stenosis of the lumbar canal; patients with bilateral radicular pain; radicular pain in lumbar spine caused by other changes than disk herniation; allergy to local anesthetics, steroids; positive history of prolonged bleeding; previous lumbar spine surgery; proven inflammatory rheumatic disease; proven inflammatory bowel disease; and other infections.

### 4.3. Minimally Invasive Treatment Methods

Epidural application of steroids through a transforaminal approach to the lumbar spine (ESI TF) is a procedure in which the drug is administered into the epidural space of the lumbar spine. This procedure is performed on patients who have pain in the lumbar part of the spine with or without spreading the pain to the lower extremity. During the procedure, the patient lies in the prone position with a pillow under the lower part of the abdomen. The procedure is performed under the control of an X-ray device. Before the procedure, the skin is disinfected with an antiseptic. The skin and subcutaneous tissue are anesthetized with a local anesthetic, and then a spinal needle is placed through the skin into the epidural space. The position of the needle is determined by an X-ray device and the administration of radiographic contrast. After confirming the correct position, a solution of 40 mg of methylprednisolone and 5 mL of 0.25% levobupivacaine was then injected according to the protocol of the institution. After the procedure, participants were closely monitored and discharged home with stable vital signs.

### 4.4. Conservative Treatment Methods

Patients diagnosed with lumbar disk herniation in the form of protrusion or extrusion were admitted to the Pain Clinic for conservative treatment in the form of physical therapy. The therapeutic protocol included the application of several physical factors using a BTL device (BTL Industries Inc., Zagreb, Croatia): laser therapy with a power of 35 mW and an energy density of 4.00 J/cm^2^, transcutaneous electrical nerve stimulation (TENS) for 10 min at a pulse of 100 µs and a frequency of 200 Hz, ultrasound therapy with a intensity of 1.0 W/cm^2^ and a frequency of 1 MHz for 5 min, magnetic therapy with 31 mT/10 intensity for 10 min, and acupuncture. The therapy was performed daily for a period of 10 days, after which the patients were examined by specialists to assess the therapeutic effect.

### 4.5. Outcome Measurement

#### 4.5.1. McGill (SF-MPQ)

McGill University Pain Questionnaire—a short version consisting of 15 items (11 sensory; 4 affective) that are rated on an intensity scale as 0 = none, 1 = mild, 2 = moderate, or 3 = severe. The scores are summed up to provide a total descriptor of pain intensity from the sensory and affective items. It also includes the current pain intensity index of the standard McGill Questionnaire (MPQ) and the visual analog scale (VAS) [48].

#### 4.5.2. Oswestry Disability Index (ODI)

The Oswestry Disability Index (ODI) subsist 10 items: pain intensity, personal care, lifting, walking, sitting, standing, sleeping, social life, sexual activity, and travel. Each item is scored from 0 to 5 points each and the total score amounts to 50, and the final scores are expressed in percent. The levels of disability in the ODI were classified as minimal disability (0–20%), moderate disability (21–40%), severe disability (41–60%), paralyzing back pain (61–80%), bedridden or exaggerating symptoms (81–100%) [48].

### 4.6. Blood Samples

Blood samples were collected immediately before the start and after two weeks of conservative treatment/minimally invasive treatment and were stored in tubes for biochemistry plus 4 mL at +4 until centrifugation in the Laboratory for Translational Medicine of the Faculty of Dental Medicine and Health Osijek. Samples were then centrifuged at 1000 rcf/10 min, and the serum was then aliquoted into separate sterile tubes and stored in a refrigerator at −80 °C until laboratory analysis.

### 4.7. Luminex

Customized ProcartaPlex Multiplex Immunoassays (Cat. number: PPXS.05-MXDJZVJ, Lot number: 438145-000) from eBioscience (Invitrogen™, Thermo Fisher Scientific, Bender MedSystems GmbH Campus Vienna Biocenter 2 A-1030 Vienna, Austria) were used to analyze pro-inflammatory cytokines (interleukin 1β (IL-1β) (calibration range 0.31–1270 pg/mL), interleukin 8 (IL-8) (calibration range 0.18–730 pg/mL)and tumor necrosis factor alpha (TNF-α) (calibration range 0.29–3640 pg/mL) and cytokines with dual roles—which can act both pro-inflammatory and anti-inflammatory—interferon gamma (IFN-γ) (calibration range 1.23–5030 pg/mL) and interleukin 6 (IL-6) (calibration range 0.79–3250 pg/mL). The principle is established on beads for protein apprehension and quantification according to the use of Luminex^®^ xMAP^®^ (multi-analyte profiling) (Bio-Techne GmbH Borsigstrasse 7A, 65205 Wiesbaden, Germany). The entire protocol was performed according to the manufacturer’s instructions (Affymetrix Inc., Vienna, Austria). The plate was read in a Luminex^®^ xMAP^®^ instrument (Bio-Techne GmbH, Wiesbaden, Germany). The levels of the tested analytes were determined using ProcartaPlex Analyst v 1.0. eBioscience, Affymetrix, Vienna, Austria) and expressed in picograms per milliliter.

### 4.8. Statistical Methods

Categorical variables were expressed as absolute and relative frequencies. Differences between categorical variables were assessed with Fisher’s exact test. The normality of the distribution of continuous data was assessed with the Shapiro–Wilk test, and due to the small sample, non-parametric methods were applied. Continuous data are expressed as median and interquartile range (IQR). Differences between two independent groups of continuous variables were analyzed with the Mann–Whitney U test, while within-group differences between measurements were assessed with the Wilcoxon signed-rank test. Correlations were assessed by Spearman’s rank correlation coefficient (ρ). All *p*-values were two-tailed, and the level of statistical significance was set at α = 0.05. Statistical analyses were performed using Statistical analysis was performed using the Statistica 10.0 program (StatSoft, Tulsa, OK, USA).

### 4.9. Ethics Committee

The study was conducted following the Declaration of Helsinki. All procedures involving the study participants were approved by the Ethics Committee of the University Hospital Osijek (No. R1-6730/2024.) and the Ethics Committee of the Faculty of Dental Medicine and Health, University of Josip Juraj Strossmayer in Osijek (No. 2158/97-97-10-24-50; Class No. 602-01/24-12/02).

Written informed consent was registered via the platform Clinical Trial (clinicaltrials.gov; Title: Immune Response During the Conservative and Minimal Invasive Treatment of Pain Caused by Lumbar Disc Herniation, Clinical Trials Number (NCT06545812), Initial Release 23 July 2024, Last Release 27 August 2025) to publish their data in this paper.

### 4.10. Study Limitations

Limitations of this study include a small sample size, lack of an untreated control group, and lack of long-term follow-up. Instead of an untreated control group, comparison was made between the two treatment modalities. Additional limitations included a lack of specific pain measurements and data on environmental factors such as smoking, which may have contributed to degenerative changes. Also, the multimodal conservative treatment approach did not specify which type of therapy altered serum levels. Future studies of larger sample sizes and longer follow-up periods are needed.

## 5. Conclusions

Comparison of conservative and minimally invasive treatments for lumbar disk herniation revealed distinct inflammatory and anti-inflammatory mechanisms. A positive correlation was observed between pain, disability, and INF-γ, and a negative correlation between TNF-α and IL-6 following minimally invasive therapy. Conservative treatment showed a tendency of short-term decrease in IL-1β and IL-8. Given the two-week follow-up, findings are preliminary; larger, long-term studies are needed to confirm cytokine-level changes and clinical outcomes. 

## Figures and Tables

**Table 1:** Sociodemographic characteristics of the participants (*n* = 28).

	Number (%) of Participants			p *
	Conservative Treatment (*n* = 14)	ESI TF (*n* = 14)	Total (*n* = 28)	
Gender				
Male	3 (21)	4 (29)	7 (25)	>0.99
Female	11 (79)	10 (71)	21 (75)	
Marital status				
Married	10 (71)	10 (71)	20 (71)	>0.99
Divorced	2 (14)	1 (7)	3 (11)	
Single	1 (7)	1 (7)	2 (7)	
Widower	1 (7)	1 (7)	2 (7)	
Education				
Primary school	0	3 (21)	3 (11)	0.40
High school	12 (86)	9 (64)	21 (75)	
Bachelor’s degree	1 (7)	1 (7)	2 (7)	
Master’s degree	1 (7)	1 (7)	2 (7)	
Working status				
Employed	7 (50)	4 (29)	11 (39)	0.09
Employed, but currently on sick leave	1 (7)	1 (7.1)	2 (7)	
Employed, but on long-term sick leave	0	3 (21)	3 (11)	
Unemployed	0	3 (21)	3 (11)	
Retired	6 (43)	3 (21)	9 (32,1)	

* Fisher’s exact test. ESI TF = transforaminal epidural steroid injection.

**Table 2:** Changes in SF-MPQ domains following conservative treatment and transforaminal epidural steroid injection.

	Median (Interquartile Range)				*p* *
	Conservative Treatment (*n* = 14)	*p* ^†^	ESI TF (*n* = 14)	*p* ^†^	
Sensory domain					
Baseline	16.5 (6.8–18.8)	0.41	16 (8.75–25.5)	0.10	0.27
After 2 weeks	10.5 (5.75–16.5)		12 (8–22.75)		0.38
Affective domain					
Baseline	4 (2–6)	0.65	9 (1.75–11.25)	0.33	0.09
After 2 weeks	3 (0–5.25)		4.5 (2.75–10)		0.12
Pain in the past week					
Baseline	60 (48.25–68.25)	0.02	70 (67.75–79.25)	0.02	0.04
After 2 weeks	47.5 (30–58.5)		50 (37.5–60)		0.75
Current pain intensity [1(none)–5 (severe)]					
Baseline	2 (2–3)	0.01	3 (2–3)	0.13	0.49
After 2 weeks	2 (1.75–2.25)		2 (1.75–3)		0.67

* Mann–Whitney U test (between groups); ^†^ Wilcoxon test (between measurements). ESI TF = transforaminal epidural steroid injection.

**Table 3:** Oswestry disability index (ODI) scores in patients treated with conservative therapy or transforaminal epidural steroid injection at baseline and after two weeks.

ODI Total	Median (Interquartile Range)				*p* *
	Conservative Treatment (*n* = 14)	*p* ^†^	ESI TF (*n* = 14)	*p* ^†^	
ODI total					
Baseline	30 (15–37)	0.06	40 (35.5–53)	0.008	0.006
After 2 weeks	34 (21–41.5)		36 (27–45.5)		0.46

* Mann–Whitney U test (between groups); ^†^ Wilcoxon test (between measurements). ESI TF = transforaminal epidural steroid injection.

**Table 4:** Correlation of cytokines in groups by measurement point.

	Spearman’s Correlation Coefficient Rho (*p* Value)				
	IFN-γ (pg/mL)	IL-1β (pg/mL)	IL-6 (pg/mL)	Il-8 (pg/mL)	TNF-α (pg/mL)
**Conservative Treatment**					
	**Baseline**				
IFN-γ (pg/mL)	-				
IL-1β (pg/mL)	−0.204 (0.50)	-			
IL-6 (pg/mL)	0.515 (0.07)	0.060 (0.85)	-		
IL-8 (pg/mL)	−0.120 (0.70)	0.280 (0.35)	0.274 (0.36)	-	
TNF-α (pg/mL)	−0.528 (0,06)	−0.047 (0.88)	−0.712 (0.006)	−0.104 (0.74)	-
	**After 2 weeks**				
IFN-γ (pg/mL)	-				
IL-1β (pg/mL)	0.587 (0.03)	-			
IL-6 (pg/mL)	0.337 (0.26)	0.198 (0.52)	-		
IL-8 (pg/mL)	0.127 (0.68)	−0.234 (0.44)	0.260 (0.39)	-	
TNF-α (pg/mL)	−0.146 (0.63)	0.063 (0.84)	−0.025 (0.94)	−0.158 (0.61)	-
**ESI TF**					
	**Baseline**				
IFN-γ (pg/mL)	-				
IL-1β (pg/mL)	0.772 (0.001)	-			
IL-6 (pg/mL)	0.159 (0.59)	0.273 (0.35)	-		
IL-8 (pg/mL)	0.210 (0.47)	0.263 (0.36)	0.505 (0.07)	-	
TNF-α (pg/mL)	0.265 (0.36)	0.464 (0.09)	0.108 (0.71)	0.153 (0.60)	-
	**After 2 weeks**				
IFN-γ (pg/mL)	-				
IL-1β (pg/mL)	0.425 (0.13)	-			
IL-6 (pg/mL)	0.432 (0.12)	0.183 (0.53)	-		
IL-8 (pg/mL)	−0.024 (0.93)	−0.135 (0.65)	−0.305 (0.29)	-	
TNF-α (pg/mL)	0.326 (0.26)	0.501 (0.07)	−0.016 (0.96)	0.040 (0.89)	-

## Data Availability

The data presented in this study are available from the corresponding author upon request.

## Supplementary Materials

The following supporting information can be downloaded at https://www.mdpi.com/article/10.3390/ijms262210830/s1.

## Abbreviations

The following abbreviations are used in this manuscript:

LDH	lumbar disk herniation
IVD	intervertebral disk
IL-1β	interleukin-1 beta
TNF-α	tumor necrosis factor-α
MMP	matrix metalloproteinase
IFN -γ	interferon- gamma
IL-8	interleukin 8
IL-6	interleukin 6
MCP-1	monocyte chemoattractant protein-1
ESI TF	transforaminal Epidural Steroid Injection
IFC	interferential Current
TENS	transcutaneous electrical nerve stimulation
SF-MPQ	short form McGill pain questionnaire
ODI	Oswestry low back pain questionnaire
VAS	visual analog scale
SCI	spinal cord injury
