# Supplementary material for: Biological Mechanisms of Pain Management in Lumbar Disk Herniation: Focus on Cytokine Correlations and Therapeutic Approaches
Source: Int J Mol Sci. 2025 Nov 7;26(22):10830. doi: 10.3390/ijms262210830 (PMC12652388; doi:10.3390/ijms262210830)
Supplement: Supplementary file 1 [file ijms-26-10830-s001.zip › ijms-3933415-supplementary.pdf]

Supplementary Table S1. Levels of inflammatory and anti-inflammatory response before and after treatment

	Median (interquartile range)				
	Conservative Treatment (n = 14)	$p^{†}$	ESI TF (n = 14)	$p^{†}$	p^{*}
Age (years)	59.5 (54.8 - 67)		52 (38.8 – 61.3)		0.05
IFN-γ					
Baseline	1.59 (1.59 – 1.61)	0.29	1.59 (1.58 – 1.62)	0.81	0.26
After 2 weeks	1.59 (1.58 – 1.62)		1.6 (1.59 – 1.63)		0.53
IL-1β					
Baseline	0.43 (0.4 – 0.44)	0.14	0.4 (0.38 – 0.44)	0.50	0.38
After 2 weeks	0.41 (0.4 – 0.42)		0.43 (0.39 – 0.46)		0.43
IL-6					
Baseline	1.32 (1.28 – 1.39)	0.58	1.32 (1.3 – 1.44)	0.51	0.46
After 2 weeks	1.32 (1.28 – 1.36)		1.33 (1.28 – 1.53)		0.61
IL-8					
Baseline	0.99 (0.28 – 1.76)	0.14	0.44 (0.36 – 0.63)	0.16	0.53
After 2 weeks	0.43 (0.3 – 0.82)		0.54 (0.38 – 0.68)		0.66
TNF-α					
Baseline	0.44 (0.38 – 0.52)	0.56	0.41 (0.37 – 0.46)	0.58	0.54
After 2 weeks	0.45 (0.41 – 0.5)		0.43 (0.38 – 0.49)		0.26

*Mann Whitney U test; †Wilcoxon test; ESI TF= transforaminal epidural steroid injection.

Supplementary Table S2. Correlation of cytokines in groups by measurement point with SF-MPQ

SF-MPQ	Spearman's correlation coefficient Rho (*p*-value)				
	INF gamma	IL-1beta	IL-6	Il-8	TNF alpha
**Conservative Treatment**	**Baseline**				
Sensory domain	-0.055 (0.86)	0.067 (0.83)	0.285 (0.34)	0.006 (0.99)	-0.458 (0.12)
Affective domain	0.085 (0.78)	-0.176 (0.57)	0.480 (0.10)	0.123 (0.69)	-0.296 (0.33)
Pain in the past week	-0.243 (0.42)	-0.560 (0.05)	-0.045 (0.89)	-0.228 (0.45)	-0.106 (0.73)
Current pain intensity	-0.499 (0.08)	-0.471 (0.10)	-0.252 (0.41)	0.007 (0.98)	0.166 (0.59)
	**After 2 weeks**				
Sensory domain	0.162 (0.60)	-0.255 (0.40)	0.055 (0.86)	0.342 (0.25)	-0.215 (0.48)
Affective domain	-0.212 (0.49)	-0.491 (0.09)	0.020 (0.95)	0.150 (0.63)	-0.256 (0.40)
Pain in the past week	0.065 (0.83)	-0.166 (0.59)	0.150 (0.63)	0.256 (0.40)	-0.291 (0.33)
Current pain intensity	-0.411 (0.16)	-0.487 (0.09)	0.064 (0.84)	0.278 (0.36)	-0.216 (0.48)
**ESI TF**	**Baseline**				
Sensory domain	-0.042 (0.89)	0.333 (0.24)	-0.159 (0.59)	0.249 (0.39)	0.330 (0.25)
Affective domain	-0.068 (0.82)	0.248 (0.39)	-0.061 (0.84)	0.360 (0.21)	0.091 (0.76)
Pain in the past week	0.238 (0.41)	0.235 (0.42)	-0.241 (0.41)	-0.238 (0.41)	-0.078 (0.79)
Current pain intensity	0.561 (0.04)	0.425 (0.13)	0.061 (0.84)	0.218 (0.45)	0.347 (0.22)
	**After 2 weeks**				
Sensory domain	-0.204 (0.48)	-0.204 (0.49)	-0.055 (0.85)	0.070 (0.81)	-0.072 (0.81)
Affective domain	0.008 (0.98)	-0.135 (0.65)	0.004 (0.99)	0.051 (0.86)	0.028 (0.92)
Pain in the past week	0.218 (0.46)	0.088 (0.76)	0.336 (0.24)	-0.038 (0.90)	-0.428 (0.13)
Current pain intensity	0.270 (0.35)	0.409 (0.15)	0.385 (0.17)	-0.251 (0.39)	-0.041 (0.89)

Supplementary Table S3. Correlation of cytokines in groups by measurement point with ODI

	Spearman's correlation coefficient Rho (*p*-value)				
	INF gamma	IL-1beta	IL-6	Il-8	TNF alpha
**Conservative Treatment**	**Baseline**				
Total ODI	-0.184 (0.55)	-0.110 (0.72)	0.057 (0.85)	0.333 (0.27)	-0.051 (0.87)
	**After 2 weeks**				
Total ODI	-0.037 (0.90)	-0.052 (0.87)	0.227 (0.46)	0.116 (0.71)	-0.348 (0.24)
**ESI TF**	**Baseline**				
Total ODI	-0.086 (0.77)	-0.009 (0.98)	0.304 (0.29)	0.522 (0.06)	0.215 (0.46)
	**After 2 weeks**				
Total ODI	0.582 (0.03)	0.210 (0.47)	0.352 (0.22)	0.095 (0.75)	-0.317 (0.27)
